# A chromosomal reference genome sequence for the malaria mosquito, *Anopheles nili*, Theobald, 1904

**DOI:** 10.12688/wellcomeopenres.23198.1

**Published:** 2024-10-16

**Authors:** Sandrine Eveline Nsango, Diego Ayala, Jean-Pierre Agbor, Harriet F. Johnson, Haynes Heaton, Martin G. Wagah, Joanna C Collins, Ksenia Krasheninnikova, Sarah E. Pelan, Damon-Lee B. Pointon, Ying Sims, James W. Torrance, Alan Tracey, Marcela Uliano-Silva, Jonathan M.D. Wood, Katharina von Wyschetzki, Wellcome Sanger Institute Scientific Operations: Sequencing Operations, Shane A. McCarthy, Daniel E. Neafsey, Alex Makunin, Mara K.N. Lawniczak

**Affiliations:** 1 Faculte de Medecine et des Sciences Pharmaceutiques, Universite de Douala, Douala, Littoral, Cameroon; 2 MIVEGEC, Univ. Montpellier, CNRS, IRD, Montpellier, France; 3 Medical Entomology Unit, Institut Pasteur de Madagascar, Antananarivo, Antananarivo Province, Madagascar; 4 Scientific Operations, Wellcome Sanger Institute, Hinxton, England, UK; 5 Auburn University, Auburn, Alabama, USA; 6 Tree of Life, Wellcome Sanger Institute, Hinxton, England, UK; 7 University of Cambridge Department of Genetics, Cambridge, England, UK; 8 Department of Immunology and Infectious Diseases, Harvard T.H. Chan School of Public Health, Boston, Massachusetts, USA; 9 Infectious Disease and Microbiome Program, Broad Institute, Cambridge, Massachusetts, USA

**Keywords:** Anopheles nili, African malaria mosquito, genome sequence, chromosomal

## Abstract

We present a genome assembly from an individual male *Anopheles nili* (the malaria mosquito; Arthropoda; Insecta; Diptera; Culicidae), from a wild population in Cameroon. The genome sequence is 195 megabases in span. Most of the assembly is scaffolded into three chromosomal pseudomolecules with the X sex chromosome assembled. The complete mitochondrial genome was also assembled and is 15.4 kilobases in length.

## Species taxonomy

Animalia; Arthropoda; Insecta; Diptera; Culicidae; Anophelinae; Anopheles; *Anopheles nili*; Theobald, 1904 (NCBI txid:185578).

## Background

*Anopheles nili* (Theobald, 1904) is a member of the *Anopheles nili* group, which includes three additional described species: *An. carnevalei*, *An. somalicus,* and *An. ovengensis* ^1, 2^. These four species exhibit slight morphological differences at the larval and adult stages ^3^; however, they are usually distinguished by molecular methods ^4^. *Anopheles nili* is the most important malaria vector in this group, with a wide geographical distribution, populating the humid savannas and degraded forested areas across West, Central and East Africa ^5, 6^. The larva of this mosquito usually breeds in streams and rivers with floating vegetation ^1, 7^. *Anopheles nili* exhibit a host preference for humans, although it can also feed on other alternative hosts ^8^. A different trophic behaviour has been observed between habitats: while forest populations prefer to bite indoors, savanna populations feed preferentially outdoors ^9, 10^. *Anopheles nili* is considered a major malaria vector by its contribution to transmission across many African countries, with naturally infective bites rate reaching up to 200/person/year ^11– 14^. The absence of insectary colonies challenges the study of this species and to date, no study has been conducted to evaluate its susceptibility to insecticides. Therefore, despite its major role in malaria transmission, key aspects of the biology and ecology of this species remain largely unknown.

The occurrence of morphologically similar species with limited role in malaria transmission fomented efforts to develop molecular species identification. To this end, several authors focused on the sequence variations of the ribosomal second internal transcribed spacers (ITS2) to develop PCR-based protocols to distinguish species within the group ^4^. A multilocus enzyme analysis also exists to detect specific differences among members of the *An. nili* group ^15^. Other molecular markers, such as microsatellites ^16^, revealed genetic heterogeneity within *An nili* populations in equatorial forest environments ^17, 18^. A high-resolution cytogenetic map within *An. nili* populations revealed two polymorphic inversions on the 2R chromosomal arm that have a potential role in local adaptation ^19^. Microsatellite markers were further mapped to the polytene chromosomes and a partial scaffold-level genome assembly was generated ^20^. In summary, some tools and genetic data exist, but given the importance of the species in malaria transmission, a high quality reference genome is warranted. *Anopheles nili* is a key species contributing to malaria transmission in forested areas of Africa and the development of genomic resources is essential to better characterise vector competence, host preference, and insecticide resistance towards improving vector control in Africa.

Here, as part of the Anopheles Reference Genomes Project (PRJEB51690), we present a chromosomally complete genome sequence for *Anopheles nili*, based on a single male specimen, collected in Mbébé, Cameroon. Importantly, this reference genome was created from specimens shipped at room temperature from Cameroon to the UK, which took longer than one week to arrive. This was achieved through lightly squishing each shipped specimen in excess ethanol (e.g. one mosquito per 1.5 mL Eppendorf tube filled with 100% ethanol). Light squishing accompanying the preservative protects the length of the DNA molecules required for long read sequencing ^21^.

## Genome sequence report

The genome was sequenced from a single male *Anopheles nili* reared from a female mosquito collected in Mbébé, Cameroon (4.173, 11.073). A total of 48-fold coverage in Pacific Biosciences single-molecule HiFi long reads (N50 9.498 kb) and 93-fold coverage in 10X Genomics read clouds were generated. Primary assembly contigs were scaffolded with chromosome conformation Hi-C data from an unrelated female mosquito. Manual assembly curation corrected 56 missing joins or misjoins, reducing the scaffold number by 25.1%.

**Figure 1.**
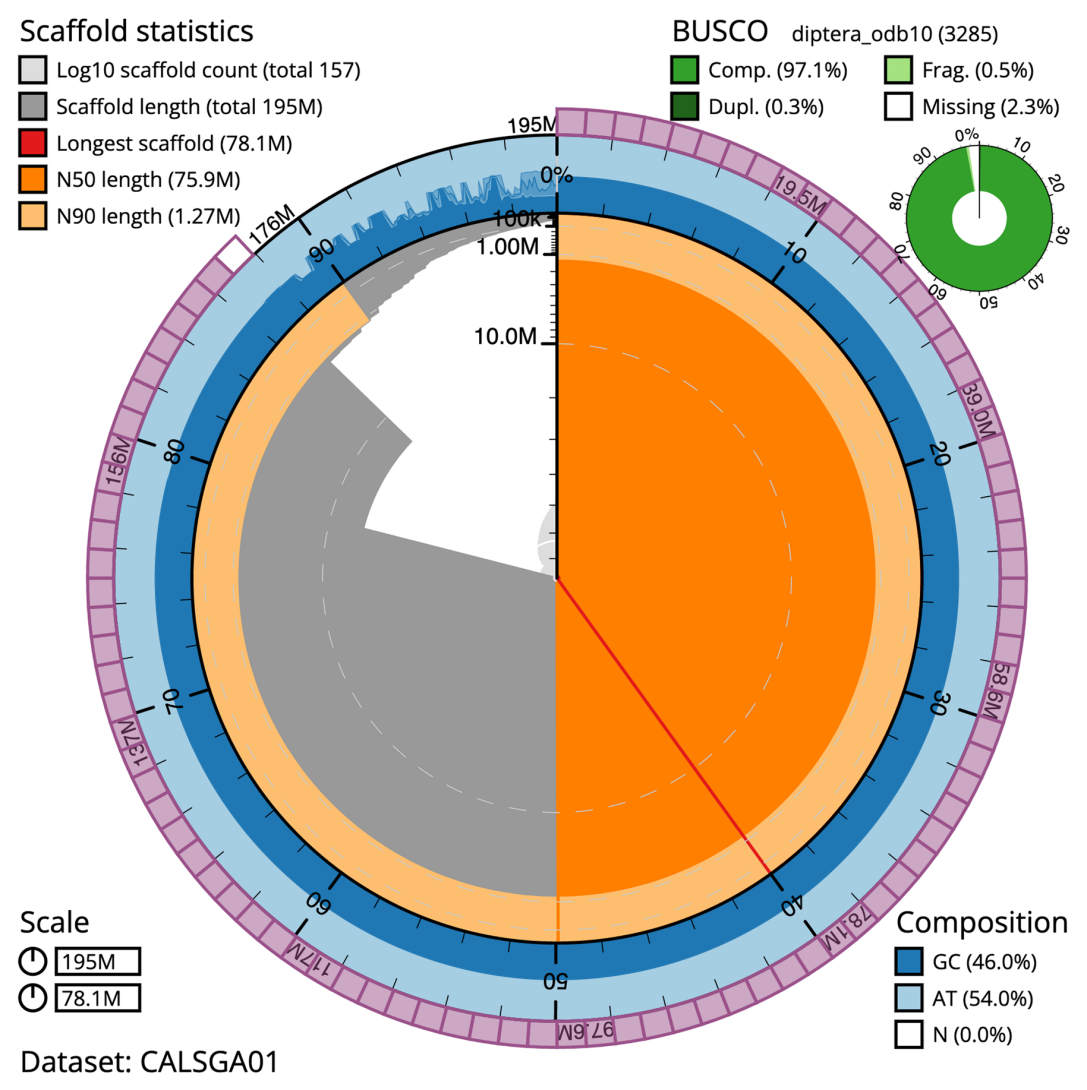
Snail plot summary of assembly statistics for *Anopheles nili* assembly idAnoNiliSN_F5_01. The main plot is divided into 1,000 size-ordered bins around the circumference with each bin representing 0.1% of the 195,236,048 bp assembly. The distribution of sequence lengths is shown in dark grey with the plot radius scaled to the longest sequence present in the assembly (78,106,464 bp, shown in red). Orange and pale-orange arcs show the N50 and N90 sequence lengths (75,938,266 and 1,265,936 bp), respectively. The pale grey spiral shows the cumulative sequence count on a log scale with white scale lines showing successive orders of magnitude. The blue and pale-blue area around the outside of the plot shows the distribution of GC, AT and N percentages in the same bins as the inner plot. A summary of complete, fragmented, duplicated and missing BUSCO genes in the diptera_odb10 set is shown in the top right. An interactive version of this figure is available at https://blobtoolkit.genomehubs.org/view/idAnoNiliSN_F5_01/dataset/CALSGA01/snail.

**Figure 2.**
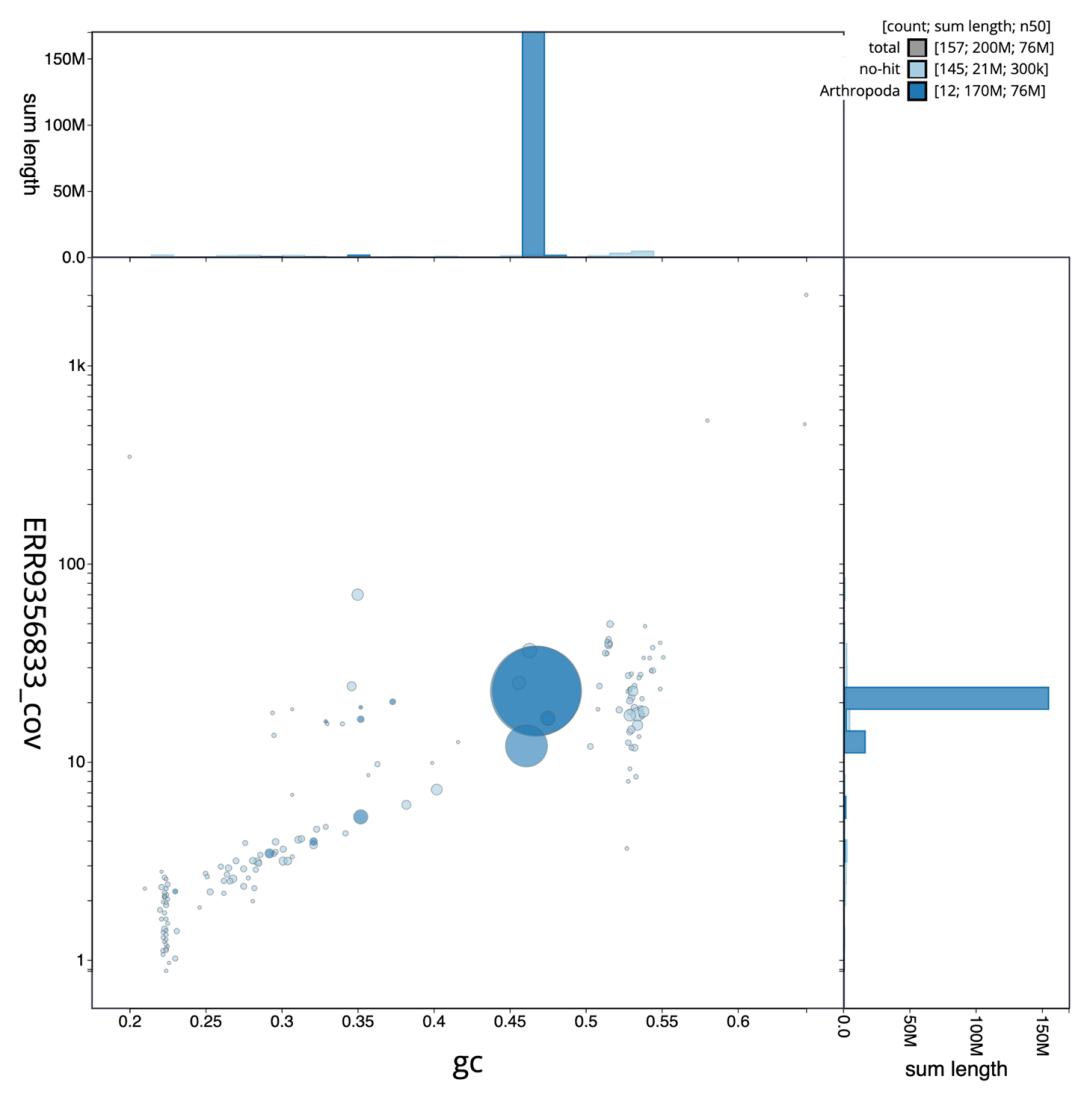
Blob plot of base coverage in a subset of idAnoNiliSN_F5_01 10x linked reads against GC proportion for *An. nili* assembly idAnoNiliSN_F5_01. Chromosomes are coloured by phylum. Circles are sized in proportion to chromosome length. Histograms show the distribution of chromosome length sum along each axis. An interactive version of this figure is available at https://blobtoolkit.genomehubs.org/view/idAnoNiliSN_F5_01/dataset/CALSGA01/blob.

**Figure 3.**
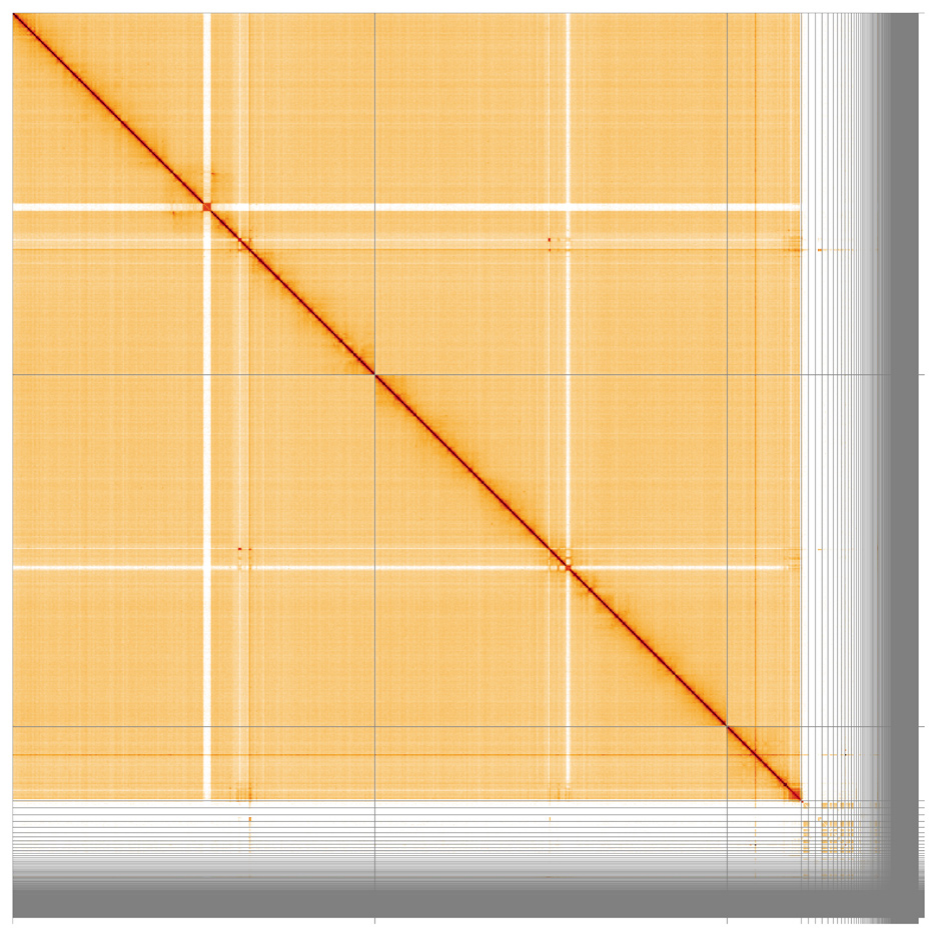
Hi-C contact map for genome assembly of *An. nili*, idAnoNiliSN_F5_01. Visualised in HiGlass. Chromosomes order: 2RL, 3RL, X, then remaining scaffolds. Signal from Y-linked scaffolds is weak because while the individual used for PacBio was male, the individual used for Hi-C was female. The white regions in the autosomes are caused by repetitive regions with lower read mapping. The interactive Hi-C map can be viewed at https://genome-note-higlass.tol.sanger.ac.uk/l/?d=JFXN0-4tSx6yNkF8v9PnSg.

**Figure 4.**
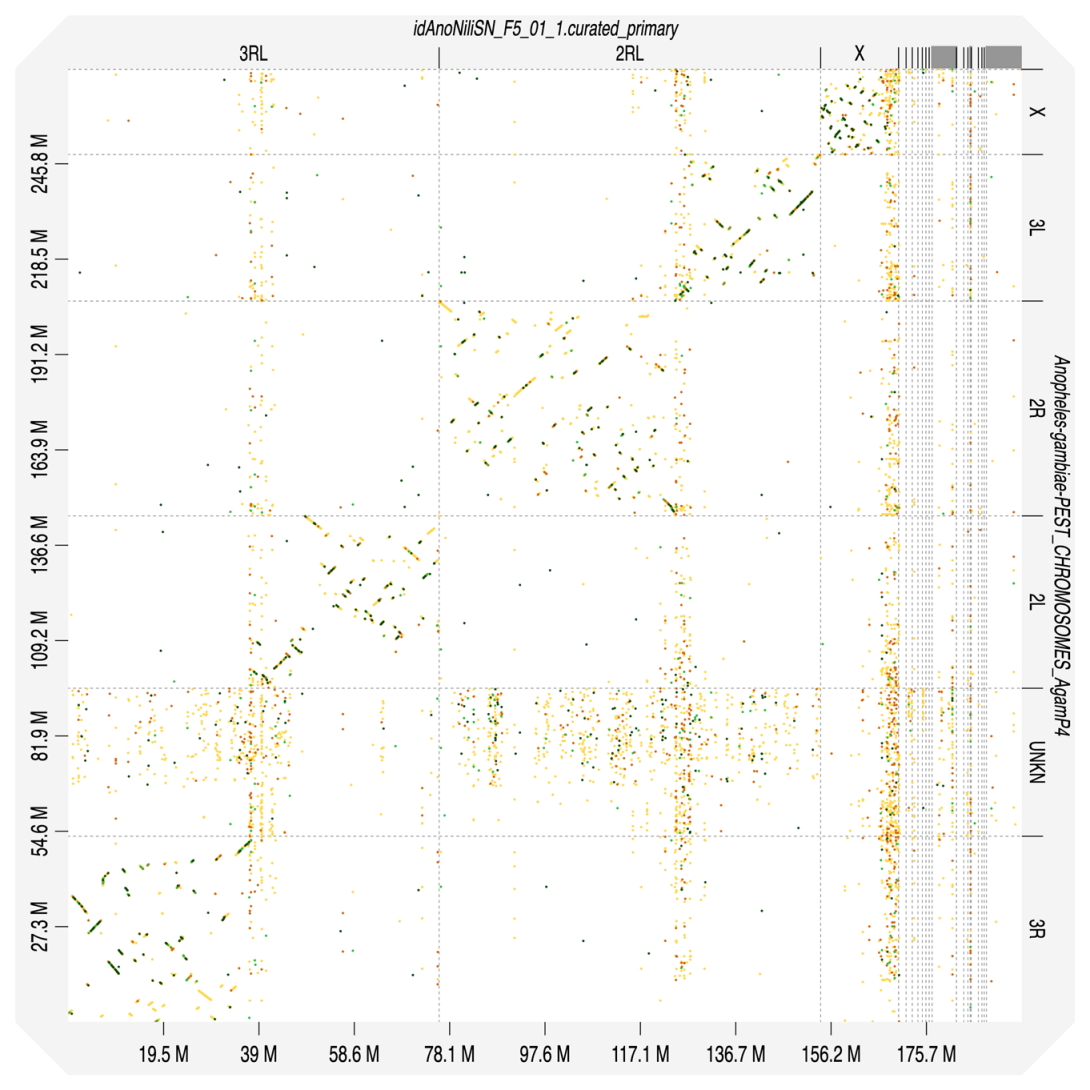
Alignment dotplot between genome assemblies of *An. nili* (idAnoNiliSN_F5_01) and *An. gambiae*, AgamP4 (PEST). Visualised in DGenies. Chromosome arms correspondence (nili-gambiae): 2R-2R, 2L-3L, 3R-3R, 3L-2L in agreement with 19.

The final assembly has a total length of 195 Mb in 157 sequence scaffolds with a scaffold N50 of 75.938 Mb ( Table 1). The snail plot in Figure 1 provides a summary of the assembly statistics, while the distribution of assembly scaffolds on GC proportion and coverage is shown in Figure 2. 87.09% of the assembly sequence was assigned to three chromosomal-level scaffolds, representing two autosomes and the X sex chromosome ( Figure 3; Table 2). Chromosomes were numbered and oriented using synteny to the *An. gambiae* PEST strain assembly AgamP3 ^22^ (accession GCF_000005575.2) with chromosome arms homologies established previously by comparative cytogenetics ^19^ ( Figure 4). The assembly has a BUSCO 5.3.2 ^23^ completeness of 97.4% using the diptera_odb10 reference set. While not fully phased, the assembly deposited is of one haplotype and also includes the circular mitochondrial genome. Contigs corresponding to the second haplotype have also been deposited.

**Table 1.** Genome data for *Anopheles nili*, idAnoNiliSN_F5_01.

*Project accession data*	
Assembly identifier	idAnoNiliSN_F5_01
Species	*Anopheles nili*
Specimen	idAnoNiliSN-F5_01
NCBI taxonomy ID	185578
BioProject	PRJEB53353
BioSample ID	ERS10527376
Isolate information	male, whole organism
*Raw data accessions*	
PacificBiosciences SEQUEL II	ERR9439508
10X Genomics Illumina	ERR9356833, ERR9356834, ERR9356835, ERR9356836
Hi-C Illumina	ERR11547908
PolyA RNA-Seq Illumina	ERR9356837, ERR9356838
*Genome assembly*	
Assembly accession	GCA_943737925
*Accession of alternate haplotype*	GCA_943737935
Span (Mb)	195.236
Number of contigs	257
Contig N50 length (Mb)	37.445
Number of scaffolds	157
Scaffold N50 length (Mb)	75.938
Longest scaffold (Mb)	78.106
BUSCO * genome score	C:97.4%[S:97.1%,D:0.3%], F:0.5%,M:2.3%,n:3285

* BUSCO scores based on the diptera_odb10 BUSCO set using BUSCO 5.3.2. C=complete [S=single copy, D=duplicated], F=fragmented, M=missing, n=number of orthologues in comparison. A full set of BUSCO scores is available at https://blobtoolkit.genomehubs.org/view/idAnoNiliSN_F5_01/dataset/CALSGA01/busco.

**Table 2.** Chromosomal pseudomolecules in the genome assembly of *An. nili*, idAnoNiliSN_F5_01.

INSDC accession	Chromosome	Size (Mb)	Count	Gaps
**OX031310.1**	2RL	78.106	1	19
**OX031311.1**	3RL	75.938	1	2
**OX031312.1**	X	15.968	1	70
**OX031313.1**	MT	0.015	1	0
	X Unlocalised	11.122	53	4
	Unplaced	14.086	100	5

**Figure 5.**
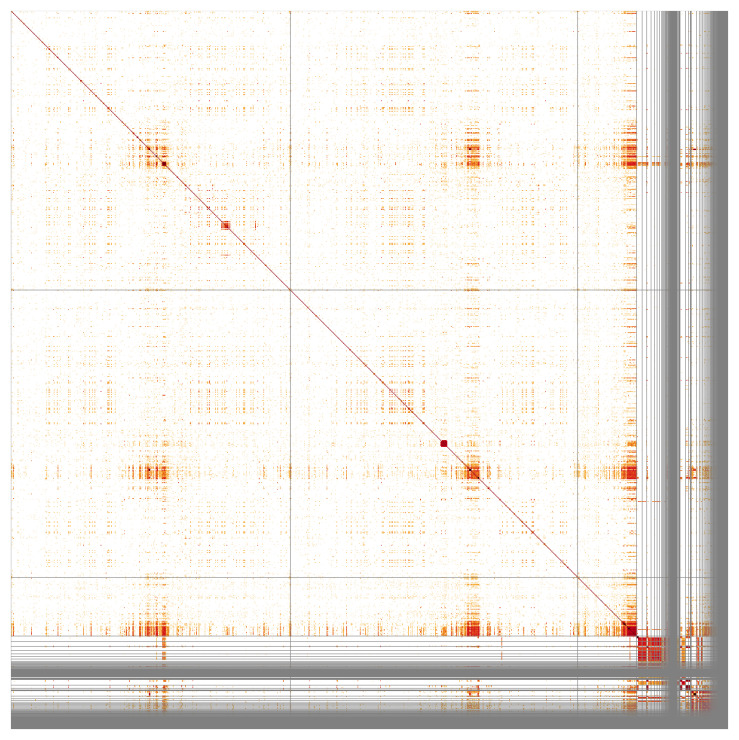
Sequence similarity heatmap for genome assembly of *An. nili*, idAnoNiliSN_F5_01. Produced with StainedGlass, visualised in HiGlass. Chromosomes order: 3RL, 2RL, X, followed by the remaining scaffolds. Darker colours represent higher sequence similarity, notably at pericentric and intercalary heterochromatin as well as in unassembled scaffolds.

Chromosome arms, candidate centromere sequences, and the rDNA region were delineated based on the presence of characteristic tandem repeat arrays ( Figure 5; Table 3). Candidate centromere regions in 2RL and 3RL comprised 547bp tandem repeat blocks interspersed with less regular tandem repeat blocks with longer units. Predicted centromere locations agree well with synteny to *An. gambiae* ( Figure 4). No plausible centromere candidate was found in the X chromosome assembly. The best candidate blocks with about 610bp repeat unit length were found in several unlocalised X-linked contigs - together with rRNA gene clusters. In addition, two megabase-scale segmental amplifications can be seen at 2RL:41.098-47.718Mbp and 3RL:41.120-42.112 Mbp as light crosses in Hi-C plot due to low read mapping quality ( Figure 3) and darker squares of high sequence identity in Figure 5.

**Table 3.** Chromosome arms in the genome assembly of *An. nili*, idAnoNiliSN_F5_01.

Chromosome	Start	End	Chromosome arm
**2RL**	1	48,746,711	2R
**2RL**	49,251,442	78,106,464	2L
**3RL**	1	37,445,156	3R
**3RL**	37,771,719	75,938,266	3L
**X**	1	15,967,621	X

Gene annotation was performed with the NCBI Eukaryotic Genome Annotation Pipeline and is available in RefSeq ^24^ under the accession GCF_943737925.1. A total of 12,354 genes were predicted, including 11,009 protein-coding genes and 1,270 non-coding RNAs. The genome assembly and gene annotations are hosted on VectorBase, www.vectorbase.org ^25^ under the identifier AnilCM1.

## Methods

### Sample acquisition and nucleic acid extraction

*Anopheles nili* offspring were reared from a wild caught gravid female collected from Mbébé, Cameroon (4.173, 11.073) by Sandrine Eveline Nsango. A single male idAnoNiliSN-F5_01 was used for Pacific BioSciences and 10x genomics, unrelated wild-caught female idAnoNiliSN-N21 was used for Arima Hi-C.

For high molecular weight (HMW) DNA extraction one whole insect (idAnoNiliSN-F5_01) was disrupted by manual grinding with a blue plastic pestle in Qiagen MagAttract lysis buffer and then extracted using the Qiagen MagAttract HMW DNA extraction kit with two minor modifications including halving volumes recommended by the manufacturer due to small sample size ( *Anopheles* mosquitoes typically weigh 2–3 mg) and running two elution steps of 100 μl each to increase DNA yield ^26^. The quality of the DNA was evaluated using an Agilent FemtoPulse to ensure that most DNA molecules were larger than 30 kb, and preferably > 100 kb. In general, single mosquito extractions ranged in total estimated DNA yield from 200 ng to 800 ng, with an average yield of 500 ng. Low molecular weight DNA was removed using an 0.8X AMpure XP purification. A small aliquot (less than ~5% of the total volume) of HMW DNA was set aside for 10X Linked Read sequencing and the rest of the DNA was sheared to an average fragment size of 12–20 Kb using a Diagenode Megaruptor 3 at speeds ranging from 27 to 30. Sheared DNA was purified using AMPure PB beads with a 1.8X ratio of beads to sample to remove the shorter fragments and concentrate the DNA sample. The concentration and quality of the sheared and purified DNA was assessed using a Nanodrop spectrophotometer and Qubit Fluorometer with the Qubit dsDNA High Sensitivity Assay kit. Fragment size distribution was evaluated by running the sheared and cleaned sample on the FemtoPulse system once more. The median DNA fragment size for *Anopheles* mosquitoes was 15 kb and the median yield of sheared DNA was 200 ng, with samples typically losing about 50% of the original estimated DNA quantity through the process of shearing and purification.

For Hi-C data generation, a separate unrelated female mosquito specimen (idAnoNiliSN-N21) was used as input material for the Arima V2 Kit according to the manufacturer’s instructions for animal tissue. This approach of using a sibling was taken in order to enable all material from a single specimen to contribute to the PacBio data generation, given that we were not always able to meet the minimum suggested guidance of starting with > 300 ng of HMW DNA from a specimen. Samples proceeded to the Illumina library prep stage even if they were suboptimal (too little tissue) going into the Arima reaction.

To assist with gene annotation, RNA was extracted from separate RNAlater (ThermoFisher) preserved mother and whole sibling male insect specimens (idAnoNiliSN-F5_M and idAnoNiliSN-F5_02, respectively) using TRIzol, according to the manufacturer’s instructions. RNA was then eluted in 50 μl RNAse-free water, and its concentration was assessed using a Nanodrop spectrophotometer and Qubit Fluorometer using the Qubit RNA Broad-Range (BR) Assay kit. Analysis of the integrity of the RNA was done using Agilent RNA 6000 Pico Kit and Eukaryotic Total RNA assay. Samples were not always ideally preserved for RNA, so qualities varied but all were sequenced anyway.

### Sequencing

We prepared libraries as per the PacBio procedure and checklist for SMRTbell Libraries using Express TPK 2.0 with low DNA input. Every library was barcoded to support multiplexing. Final library yields ranged from 20 ng to 100 ng, representing only about 25% of the input sheared DNA. Libraries from two specimens were typically multiplexed on a single 8M SMRT Cell. Sequencing complexes were made using Sequencing Primer v4 and DNA Polymerase v2.0. Sequencing was carried out on the Sequel II system with 24-hour run time and 2-hour pre-extension. A 10X Genomics Chromium read cloud sequencing library was also constructed according to the manufacturer’s instructions (this product is no longer available). Only 0.5 ng of DNA was used and only 25–50% of the gel emulsion was put forward for library prep due to the small genome size. For Hi-C data generation, following the Arima HiC V2 reaction, samples were processed through Library Preparation using a NEB Next Ultra II DNA Library Prep Kit and sequenced aiming for 100x depth. RNA libraries were created using the directional NEB Ultra II stranded kit. Sequencing was performed by the Scientific Operations core at the Wellcome Sanger Institute on Pacific Biosciences SEQUEL II (HiFi), Illumina NovaSeq 6000 (10X and Hi-C), or Illumina HiSeq 4000 (RNAseq).

### Genome assembly

Assembly was carried out with Hifiasm ^27^; haplotypic duplications were identified and removed with purge_dups ^28^. One round of polishing was performed by aligning 10X Genomics read data to the assembly with Long Ranger ALIGN, calling variants with FreeBayes ^29^. The assembly was then scaffolded with Hi-C data ^30^ using SALSA2 ^31^. The assembly was checked for contamination as described previously ^32^. Manual curation was performed using gEVAL ^33^, HiGlass ^34^ and Pretext ^35^. The mitochondrial genome was assembled using MitoHiFi ^36^, which performs annotation using MitoFinder ^37^. The genome was analysed and BUSCO scores were generated within the BlobToolKit environment ^38^. Synteny analysis was performed with D-GENIES ^39^ and minimap2 ^40^. Repetitive sequences were visualised with StainedGlass ^41^ and tandem repeats were annotated with ULTRA ^42^. Table 4 contains a list of all software tool versions used, where appropriate.

**Table 4.** Software tools used.

Software tool	Version	Source
hifiasm	0.14	27
purge_dups	1.2.3	28
SALSA2	2.2-4c80ac1	31
Long Ranger ALIGN	2.2.2	43
FreeBayes	1.3.1	29
MitoHiFi	2	36
gEVAL	N/A	33
HiGlass	1.11.6	34
PretextView	0.1.x	35
BlobToolKit	3.4.0	38
BUSCO	5.3.2	23
D-GENIES	1.4	39
minimap2	2.24	40
StainedGlass	0.5	41
ULTRA	1.0.0 beta	42

## Ethics/compliance issues

The genetic resources accessed and utilised under this project were done so in accordance with the UK ABS legislation (Nagoya Protocol (Compliance) (Amendment) (EU Exit) Regulations 2018 (SI 2018/1393)) and the national ABS legislation within the country of origin, where applicable.

## Data Availability

European Nucleotide Archive: *Anopheles nili* genome assembly, idAnoNiliSN_F5_01. Accession number PRJEB53353; https://identifiers.org/bioproject/PRJEB53353. The genome sequence is released openly for reuse. The *Anopheles nili* genome sequencing initiative is part of the Anopheles Reference Genomes project PRJEB51690. All raw sequence data and the assembly have been deposited in INSDC databases. Raw data and assembly accession identifiers are reported in Table 1.

## Author information

Members of Wellcome Sanger Institute Scientific Operations: Sequencing Operations are listed here: https://doi.org/10.5281/zenodo.12165051.

## References

[1] Gillies MT Coetzee M : A supplement to the Anophelinae of Africa south of the Sahara (Afrotropical region).The South African Institute for Medical Research,1987. Reference Source

[2] Awono-Ambene HP Kengne P Simard F : Description and bionomics of *Anopheles* ( *Cellia*) *ovengensis* (Diptera: Culicidae), a new malaria vector species of the *Anopheles nili* group from south Cameroon. *J Med Entomol.* 2004;41(4):561–568. 10.1603/0022-2585-41.4.561 15311444

[3] Coetzee M : Key to the females of Afrotropical *Anopheles* mosquitoes (Diptera: Culicidae). *Malar J.* 2020;19(1): 70. 10.1186/s12936-020-3144-9 32054502 PMC7020601

[4] Kengne P Awono-Ambene P Antonio-Nkondjio C : Molecular identification of the *Anopheles nili* group of African malaria vectors. *Med Vet Entomol.* 2003;17(1):67–74. 10.1046/j.1365-2915.2003.00411.x 12680928

[5] Antonio-Nkondjio C Simard F : Highlights on *Anopheles nili* and *Anopheles moucheti*, Malaria Vectors in Africa.In: Manguin S, editor. *Anopheles Mosquitoes: New Insights into Malaria Vectors*. Rijeka (HR): InTech,2013. 28045480

[6] Mouchet J : Biodiversité du paludisme dans le monde.John Libbey Eurotext,2004. Reference Source

[7] Ayala D Costantini C Ose K : Habitat suitability and ecological niche profile of major malaria vectors in Cameroon. *Malar J.* 2009;8: 307. 10.1186/1475-2875-8-307 20028559 PMC2805691

[8] Antonio-Nkondjio C Awono-Ambene HP Fontenille D : La presence des bovins comme hotes alternatifs peut-elle modifier le comportement trophique des vecteurs du paludisme en zone de foret? *Bull liaison doc - OCEAC.* 2009;1(1):7–12. Reference Source

[9] Antonio-Nkondjio C Awono-Ambene HP Etang J : Epidemiological importance of the *Anopheles Nili* group of malaria vectors in equatorial Villages of Cameroon; Central Africa.Bull liaison doc -OCEAC,2009;1(1):13–20. Reference Source

[10] Koffi AA Camara S Ahoua Alou LP : *Anopheles* vector distribution and malaria transmission dynamics in Gbêkê region, central Côte d’Ivoire. *Malar J.* 2023;22(1): 192. 10.1186/s12936-023-04623-1 37349819 PMC10288776

[11] Ossè RA Tokponnon F Padonou GG : Involvement of *Anopheles nili* in *Plasmodium falciparum* transmission in North Benin. *Malar J.* 2019;18(1): 152. 10.1186/s12936-019-2792-0 31036025 PMC6489317

[12] Bamou R Mbakop LR Kopya E : Changes in malaria vector bionomics and transmission patterns in the equatorial forest region of Cameroon between 2000 and 2017. *Parasit Vectors.* 2018;11(1): 464. 10.1186/s13071-018-3049-4 30103825 PMC6090627

[13] Adja AM N’goran EK Koudou BG : Contribution of *Anopheles funestus*, *An. gambiae* and *An. nili* (Diptera: Culicidae) to the perennial malaria transmission in the southern and western forest areas of Côte d’Ivoire. *Ann Trop Med Parasitol.* 2011;105(1):13–24. 10.1179/136485910X12851868780388 21294945 PMC4089788

[14] Antonio-Nkondjio C Kerah CH Simard F : Complexity of the malaria vectorial system in Cameroon: contribution of secondary vectors to malaria transmission. *J Med Entomol.* 2006;43(6):1215–1221. 10.1603/0022-2585(2006)43[1215:cotmvs]2.0.co;2 17162956

[15] Awono-Ambene HP Simard F Antonio-Nkondjio C : Multilocus enzyme electrophoresis supports speciation within the *Anopheles nili* group of malaria vectors in Cameroon. *Am J Trop Med Hyg.* 2006;75(4):656–658. 17038689

[16] Berthomieu A Kengne P Awono-Ambene P : Isolation and characterization of microsatellite DNA markers in the malaria vector *Anopheles nili*. *Mol Ecol Notes.* 2003;3(3):394–396. 10.1046/j.1471-8286.2003.00462.x

[17] Ndo C Antonio-Nkondjio C Cohuet A : Population genetic structure of the malaria vector *Anopheles nili* in sub-Saharan Africa. *Malar J.* 2010;9: 161. 10.1186/1475-2875-9-161 20540796 PMC2898787

[18] Ndo C Simard F Kengne P : Cryptic genetic diversity within the *Anopheles nili* group of malaria vectors in the equatorial forest area of Cameroon (Central Africa). *PLoS One.* 2013;8(3): e58862. 10.1371/journal.pone.0058862 23516565 PMC3597579

[19] Sharakhova MV Antonio-Nkondjio C Xia A : Cytogenetic map for *Anopheles nili*: application for population genetics and comparative physical mapping. *Infect Genet Evol.* 2011;11(4):746–754. 10.1016/j.meegid.2010.06.015 20603229 PMC3036789

[20] Peery A Sharakhova MV Antonio-Nkondjio C : Improving the population genetics toolbox for the study of the African malaria vector *Anopheles nili*: microsatellite mapping to chromosomes. *Parasit Vectors.* 2011;4: 202. 10.1186/1756-3305-4-202 22011455 PMC3222614

[21] Teltscher F Lawniczak M : Squishing insects for preservation of HMW DNA in the field.2023; [cited 16 Jan 2024]. 10.17504/protocols.io.4r3l2224jl1y/v1

[22] Sharakhova MV Hammond MP Lobo NF : Update of the *Anopheles gambiae* PEST genome assembly. *Genome Biol.* 2007;8(1): R5. 10.1186/gb-2007-8-1-r5 17210077 PMC1839121

[23] Simão FA Waterhouse RM Ioannidis P : BUSCO: assessing genome assembly and annotation completeness with single-copy orthologs. *Bioinformatics.* 2015;31(19):3210–3212. 10.1093/bioinformatics/btv351 26059717

[24] Pruitt KD Brown GR Hiatt SM : RefSeq: an update on mammalian reference sequences. *Nucleic Acids Res.* 2014;42(Database issue):D756–63. 10.1093/nar/gkt1114 24259432 PMC3965018

[25] Giraldo-Calderón GI Harb OS Kelly SA : VectorBase.org updates: bioinformatic resources for invertebrate vectors of human pathogens and related organisms. *Curr Opin Insect Sci.* 2022;50: 100860. 10.1016/j.cois.2021.11.008 34864248 PMC9133010

[26] Teltscher F Johnson H Lawniczak M : Manual extraction of High Molecular Weight DNA from single mosquitoes using the Qiagen MagAttract HMW DNA kit.2023. 10.17504/protocols.io.n92ldp6ool5b/v1

[27] Cheng H Concepcion GT Feng X : Haplotype-resolved *de novo* assembly using phased assembly graphs with hifiasm. *Nat Methods.* 2021;18(2):170–175. 10.1038/s41592-020-01056-5 33526886 PMC7961889

[28] Guan D McCarthy SA Wood J : Identifying and removing haplotypic duplication in primary genome assemblies. *Bioinformatics.* 2020;36(9):2896–2898. 10.1093/bioinformatics/btaa025 31971576 PMC7203741

[29] Garrison E Marth G : Haplotype-based variant detection from short-read sequencing. arXiv [q-bio.GN],2012. 10.48550/arXiv.1207.3907

[30] Rao SSP Huntley MH Durand NC : A 3D map of the human genome at kilobase resolution reveals principles of chromatin looping. *Cell.* 2014;159(7):1665–1680. 10.1016/j.cell.2014.11.021 25497547 PMC5635824

[31] Ghurye J Rhie A Walenz BP : Integrating Hi-C links with assembly graphs for chromosome-scale assembly. *PLoS Comput Biol.* 2019;15(8): e1007273. 10.1371/journal.pcbi.1007273 31433799 PMC6719893

[32] Howe K Chow W Collins J : Significantly improving the quality of genome assemblies through curation. *GigaScience.* 2021;10(1): giaa153. 10.1093/gigascience/giaa153 33420778 PMC7794651

[33] Chow W Brugger K Caccamo M : gEVAL - a web-based browser for evaluating genome assemblies. *Bioinformatics.* 2016;32(16):2508–2510. 10.1093/bioinformatics/btw159 27153597 PMC4978925

[34] Kerpedjiev P Abdennur N Lekschas F : HiGlass: web-based visual exploration and analysis of genome interaction maps. *Genome Biol.* 2018;19(1): 125. 10.1186/s13059-018-1486-1 30143029 PMC6109259

[35] PretextView: OpenGL Powered Pretext Contact Map Viewer.Github,2022. Reference Source

[36] Uliano-Silva M Nunes JGF Krasheninnikova K : marcelauliano/MitoHiFi: mitohifi_v2.0.2021. 10.5281/zenodo.5205678

[37] Allio R Schomaker-Bastos A Romiguier J : MitoFinder: efficient automated large-scale extraction of mitogenomic data in target enrichment phylogenomics. *Mol Ecol Resour.* 2020;20(4):892–905. 10.1111/1755-0998.13160 32243090 PMC7497042

[38] Challis R Richards E Rajan J : BlobToolKit--interactive quality assessment of genome assemblies. *G3 (Bethesda).* 2020;10(4):1361–1374. 10.1534/g3.119.400908 32071071 PMC7144090

[39] Cabanettes F Klopp C : D-GENIES: dot plot large genomes in an interactive, efficient and simple way. *PeerJ.* 2018;6: e4958. 10.7717/peerj.4958 29888139 PMC5991294

[40] Li H : Minimap2: pairwise alignment for nucleotide sequences. *Bioinformatics.* 2018;34(18):3094–3100. 10.1093/bioinformatics/bty191 29750242 PMC6137996

[41] Vollger MR Kerpedjiev P Phillippy AM : StainedGlass: interactive visualization of massive tandem repeat structures with identity heatmaps. *Bioinformatics.* 2022;38(7):2049–2051. 10.1093/bioinformatics/btac018 35020798 PMC8963321

[42] Olson D Wheeler T : ULTRA: a model based tool to detect tandem repeats. *ACM BCB.* 2018;2018:37–46. 10.1145/3233547.3233604 31080962 PMC6508075

[43] Long Ranger BASIC and ALIGN Pipelines: Software -Genome & Exome -Official 10x Genomics Support.[cited 16 Dec 2022]. Reference Source

