# Supplementary material for: A chromosomal reference genome sequence for the malaria mosquito, Anopheles nili, Theobald, 1904
Source: Wellcome Open Res. 2024 Oct 16;9:599. [Version 1] doi: 10.12688/wellcomeopenres.23198.1 (PMC12515363; doi:10.12688/wellcomeopenres.23198.1)
Supplement: Peer review history

# Reviewer report: Reviewer response for version 1

**DOI:** 10.21956/wellcomeopenres.25548.r134694

**Published:** 2025-10-11

**Authors:** Craig Wilding

**Affiliations:** 1 University College Dublin School of Biology and Environmental Science (Ringgold ID: 123708), Dublin, Leinster, Ireland; 2 Liverpool John Moores University, England, UK

This is a valuable additional high-quality *Anopheles* genome sequence that contributes both to the *Anopheles* Reference Genomes Project and more widely. The introduction presents an excellent background to the species and the utility of the generated sequence.

 In this introduction mention is made of forest and savannah forms of *A. nili*. The methods should make clear which was sampled and sequenced for this project.

 The end of the introduction states that the samples were 'squished' in ethanol and shipped to the UK. However, no additional details of this are given in the methods where this should be stated and detailed.

 It is my (potentially incorrect) understanding that Hi-C would not work on samples that have been preserved in ethanol since ethanol disrupts the nuclear structure and those proteins important for chromatin architecture. Can it therefore be made clear in the methods just how samples were preserved for Hi-C?

 The methods states "In general, single mosquito extractions ranged in total estimated DNA yield from 200 ng to 800 ng, with an average yield of 500 ng." How is this relevant when only 1 mosquito was extracted? In fact, this whole paragraph is just generic text about *Anopheles* extractions rather than specific to this specimen. This seems lazy and wrong - especially given the unique preservation techniques for these samples which would likely have implications for yield and quality. It would be very useful to have this rewritten specific to this specimen. It would be also useful if the Genome Sequence Report section commented on the utility of this preservation/shipping method for future DToL sequencing. This reference sequence is not just useful as a genome for this species but as a report on how to obtain chromosomal-level sequences from specimens preserved in ethanol and shipped at ambient. It is important therefore to be clear on the implications and outcomes of this for this genome sequence. I don't think this report does this clearly enough.

Are sufficient details of methods and materials provided to allow replication by others?

No

Is the rationale for creating the dataset(s) clearly described?

Yes

Are the datasets clearly presented in a useable and accessible format?

Yes

Are the protocols appropriate and is the work technically sound?

Yes

Reviewer Expertise:

Evolutionary genetics

I confirm that I have read this submission and believe that I have an appropriate level of expertise to confirm that it is of an acceptable scientific standard, however I have significant reservations, as outlined above.

# Reviewer report: Reviewer response for version 1

**DOI:** 10.21956/wellcomeopenres.25548.r134696

**Published:** 2025-10-11

**Authors:** Igor Sharakhov

**Affiliations:** 1 Virginia Polytechnic Institute and State University, Blacksburg, USA

The article by Nsango and coauthors present a genome assembly from an individual male *Anopheles nili *(the malaria mosquito; Arthropoda; Insecta; Diptera; Culicidae), from a wild population in Cameroon. *Anopheles nili* is considered a major malaria vector by its contribution to transmission across many African countries. Therefore, creating a high-quality genome reference is justified. The *An. nili* genome was sequenced using the PacBio HiFi, polished with 10X Genomics read data and scaffolded into three chromosomal pseudomolecules using Hi-C data obtained from a single mosquito male. The complete mitochondrial genome was also assembled. The manuscript provides sufficient details of methods and materials to allow replication by others.

 Comments:

 To better understand the data, it would be helpful to indicate where the centromeric regions are located in both the Hi-C contact map (Figure 3) and the sequence similarity heatmap (Figure 5). The centromeric regions appear to coincide with the white regions in the Hi-C contact map for the 3RL autosomes, but not the 2RL autosomes. The centromere region in the 2RL autosome Hi-C contact map looks like a light line, not a solid white line. How many kilobases do the 547 bp tandem repeat blocks occupy in the genome? Could they belong to pericentromeric heterochromatin rather than the actual centromere?

Are sufficient details of methods and materials provided to allow replication by others?

Yes

Is the rationale for creating the dataset(s) clearly described?

Yes

Are the datasets clearly presented in a useable and accessible format?

Yes

Are the protocols appropriate and is the work technically sound?

Yes

Reviewer Expertise:

Genomics, cytogenetics

I confirm that I have read this submission and believe that I have an appropriate level of expertise to confirm that it is of an acceptable scientific standard.

# Reviewer report: Reviewer response for version 1

**DOI:** 10.21956/wellcomeopenres.25548.r125978

**Published:** 2025-09-02

**Authors:** Renee Ali

**Affiliations:** 1 Molecular Microbiology and Immunology, Johns Hopkins University Bloomberg School of Public Health, Baltimore, Maryland, USA

This reference genome assembly fills a knowledge gap in genomic resources which can be useful for malaria mitigation strategies and understanding vector biology for an important malaria vector - *An. nili *in Africa. The combination of long-read sequencing and multiple supporting methods have delivered a well-supported by BUSCO genome assembly and gene annotation data. Though assembly statistics are strong, there may be gaps in assembly with respect to incomplete representation of centromeric region of the X chromosome- this may require additional specialized sequencing methods.

Are sufficient details of methods and materials provided to allow replication by others?

Yes

Is the rationale for creating the dataset(s) clearly described?

Yes

Are the datasets clearly presented in a useable and accessible format?

Yes

Are the protocols appropriate and is the work technically sound?

Yes

Reviewer Expertise:

vector biology and mosquito population genomics

I confirm that I have read this submission and believe that I have an appropriate level of expertise to confirm that it is of an acceptable scientific standard.
